# Risk Factors Associated With Placenta Previa: A Matched Case-Control Study

**DOI:** 10.7759/cureus.101157

**Published:** 2026-01-09

**Authors:** Hadia Javed, Ume Habiba, Umm E Rubab, Sana Saleem, Zaira A Khan, Labeeba Abdul Ghafoor, Sarah Karim, Syeda Misbah Noor, Maleeha Shah, Wagma Shahriyar Khan

**Affiliations:** 1 Obstetrics and Gynaecology, Ayub Teaching Hospital, Abbottabad, PAK; 2 Medicine and Surgery, Ayub Teaching Hospital, Abbottabad, PAK; 3 Medical Education, Binzhou Medical University, Yantai, CHN; 4 Obstetrics and Gynaecology, Punjab Medical College, Faisalabad, PAK; 5 Obstetrics and Gynaecology, Northwest General Hospital and Research Center, Peshawar, PAK; 6 Internal Medicine, Combined Military Hospital, Muzaffarabad, PAK; 7 Obstetrics and Gynaecology, District Headquarter (DHQ) Hospital Mishti Mela, Orakzai, PAK

**Keywords:** advanced maternal age, assisted reproductive techniques, case-control study, cesarean section, dilatation and curettage, placenta previa, risk factors

## Abstract

Introduction

Placenta praevia is a recognized pregnancy complication characterized by the placenta implanting partially or completely within the lower uterine segment, and it is associated with significant maternal and perinatal morbidity. Antepartum hemorrhage is a cardinal sign of placenta praevia, and it is unusual for a woman with placenta praevia to reach the late third trimester without vaginal bleeding. Many risk factors, i.e., age >30 years, previous caesarean section, history of dilatation and curettage, history of assisted reproductive techniques (ARTs), multiple pregnancies, and history of placenta praevia in previous pregnancies, need to be addressed so that a confirmed diagnosis can be made in the antenatal period, and women who are at increased risk for this condition can be managed or referred to a setup where proper and vigilant care is provided to both mother and baby. This study aims to identify the risk factors unique to our population and to stratify antenatal patients into high-risk and lower-risk pregnancy groups.

Methods

The study was conducted in the Obstetrical Department of Ayub Teaching Hospital, Abbottabad, Pakistan, after approval of the topic and completion of all prerequisites, for a period of three months from July 1, 2025, to September 30, 2025. Women diagnosed with placenta praevia, including low-lying placenta praevia, through ultrasound examinations after 32 completed weeks of gestation were included in the case group. A total of 30 patients were included in the case group. Similarly, 60 women with normal placental localization were included in the control group (case:control ratio, 1:2). Detailed history was taken and recorded in a predefined proforma. For qualitative data, the Chi-square test was used, and a p-value of <0.05 was considered significant.

Results

In our study, several factors were associated with placenta previa. Women older than 30 years were more likely to develop placenta previa, with nearly four times higher odds compared to younger women (odds ratio (OR) 3.92, 95% confidence interval (CI): 1.55-9.93; p = 0.0064). A history of previous cesarean section also increased the risk, with cases showing six times higher odds than controls (OR: 6.00, 95% CI: 2.28-15.77; p = 0.00037). Similarly, women who had previously undergone dilatation and curettage had over five times higher odds of placenta previa (OR: 5.20, 95% CI: 1.97-13.70; p = 0.00095), and those who conceived through ARTs were nearly four times more likely to develop the condition (OR: 3.80, 95% CI: 1.49-9.67; p = 0.0037). Notably, a previous history of placenta previa was the strongest predictor of recurrence, with affected women facing markedly higher odds of developing the condition again (OR: 11.0, 95% CI: 3.23-37.42; p = 0.0001).

Conclusion

Our study highlights several key risk factors for placenta previa, including advanced maternal age, previous cesarean section, history of dilatation and curettage, ARTs, and prior placenta previa. Identifying these factors early can help clinicians recognize women at higher risk and implement careful monitoring to prevent complications. While our findings align with larger studies, they provide additional insight by examining these associations within a focused case-control setting. Overall, these results emphasize the importance of individualized risk assessment and proactive management to improve maternal and fetal outcomes in pregnancies complicated by placenta previa.

## Introduction

Placenta previa is a pathological condition in which the placenta completely or partially obstructs the internal cervical os. According to the updated RCOG (Royal College of Obstetricians and Gynaecologists) guidelines, placenta praevia is classified into two categories: low-lying placenta and complete placenta praevia. A placenta is defined as low-lying when the placental edge is situated less than 20 mm from the internal cervical os after 16 weeks of gestation. It is considered normal when the placental margin lies 20 mm or more from the internal os. Complete placenta praevia is diagnosed when the placenta entirely covers the internal cervical os on transabdominal or transvaginal ultrasonography [1].

The previous classification of placenta previa was as follows: Grade I, or minor placenta previa, when the lower edge of the placenta is in the lower uterine segment; Grade II, when the lower edge of the placenta reaches the internal cervical os; Grade III, when the lower edge of the placenta partially covers the internal cervical os; and Grade IV, when the placenta completely covers the internal cervical os. Grades I and II were considered minor, and Grades III and IV were considered major placenta previa. The updated classification provides a clearer description of complications, such as postpartum hemorrhage (PPH), and aims to optimize the obstetrical management of placenta previa [2,3].

Placenta previa is associated with significant morbidity and mortality [4,5]. Women with this condition are at increased risk of anemia, massive haemorrhage (with the requirement for blood transfusion reported to be about 12-fold greater in cesarean sections performed for placenta praevia than in those performed for other reasons), prolonged hospitalization, venothromboembolism, PPH, and cesarean hysterectomy [6-8].

Keeping in view the complications that may arise, the antenatal woman should be classified as a high-risk pregnancy, and delivery should be undertaken in a maternity facility equipped with immediate bedside blood transfusion capabilities and within reach of an intensive care unit.

Not only the mother, but neonates born to a mother with this condition also suffer from significant morbidity, including low APGAR score, respiratory distress syndrome, prematurity, multiple neonatal intensive care unit (NICU) admissions, mechanical ventilation, anemia, and intraventricular hemorrhage [9].

Therefore, in the routine mid-anomaly fetal scan (18+6 to 21+6 weeks of gestation), the main aim is to determine placental localization [10,11]. As the lower uterine segment develops during the third trimester, apparent placental ‘migration’ occurs, resulting in the fixation of a low-lying placenta in nearly 90% of cases before term [12-14]. That is why RCOG recommends repeated ultrasound at 32 weeks of gestation to label the placenta as a persistent low-lying placenta or a persistent placenta previa. However, there is a possibility that migration of the placenta occurs after 32 weeks [14,15].

Placenta previa has a prevalence of 0.64% in Asian women and 3-4/1000 worldwide [16]. There are several risk factors for placenta previa, including previous cesarean sections, artificial reproductive techniques, increased maternal age, smoking, and many obstetrical complications, such as manual removal of the placenta and evacuation for retained products of conception [17-19]. The basic pathology behind these risk factors is unexplained.

This study aimed to identify and quantify the association between selected maternal and obstetric risk factors and placenta previa in our population, using a case-control design, in order to stratify antenatal women into high- and low-risk groups and to facilitate timely referral and appropriate obstetric management.

## Materials and methods

This study was conducted in the Obstetrical Department of Ayub Teaching Hospital, Abbottabad, Pakistan. After completing all the prerequisites and obtaining ethical approval (RC-EA-2025/193) on June 18, 2025, from the respective authorities, the study was commenced. The data were collected prospectively over a three-month period, from July 1, 2025, to September 30, 2025. Non-probability purposive sampling was employed for case selection. We used this sampling technique to deliberately include participants who met specific diagnostic and clinical criteria, rather than selecting them randomly from the general population. A total of 30 cases and 60 controls were included in the study, keeping in view the reported prevalence of placenta praevia (0.64%) and a 5% margin of error [16]. The sample size was deemed adequate to achieve the study objectives within the available resources and time frame.

All patients who had been diagnosed with placenta previa on ultrasound after 32 weeks of gestation were included in the study as the case group, irrespective of whether they had antepartum hemorrhage or not. Similarly, patients who presented with per vaginal bleeding due to other reasons were excluded, i.e., placental abruption, vasa previa, or other undetermined causes. Patients with incomplete data were also excluded from the study. Sixty antenatal patients were selected as controls, applying a 1:2 case-to-control ratio. This approach was adopted to optimize statistical efficiency, minimize potential confounding, and allow for a robust and reliable comparison with the study cohort.

In our study, parity was taken as a matching factor to reduce possible confounding related to differences in previous obstetric history. For every woman diagnosed with placenta previa, controls with a similar number of previous deliveries (whether nulliparous, primiparous, or multiparous) were included, allowing us to make a fair comparison and better understand how other factors, such as maternal age, prior cesarean section, and uterine procedures, contributed to the risk of developing placenta previa.

We collected all the information using a self-made questionnaire, where the obstetrical, gynaecological, social, and demographic history was recorded in detail (Appendix 1).

Data were analyzed using IBM SPSS Statistics for Windows, Version 20 (Released 2011; IBM Corp., Armonk, NY, USA). Descriptive statistics were used to summarize the demographic and obstetric characteristics of cases and controls. Categorical variables were compared using the Chi-square test. For each potential risk factor, odds ratios (ORs) with 95% confidence intervals (CIs) were calculated to estimate the strength of association with placenta previa, consistent with the case-control study design. A p-value of less than 0.05 was considered statistically significant.

## Results

In our study, maternal age greater than 30 years was significantly associated with placenta previa. Among the cases, 56.7% (17/30) of women were older than 30 years, compared to 25% (15/60) in the control group. The odds of developing placenta previa were nearly four times higher in women above 30 years of age (OR: 3.92, 95% CI: 1.55-9.93; p = 0.0064). Our results highlighted advanced maternal age as an independent risk factor for placenta previa.

A history of previous cesarean section was significantly more common among cases than controls: 60.0% (18/30) of cases, versus 20.0% (12/60) of controls. The odds of placenta praevia were six times higher in women with a prior cesarean section (OR 6.00, 95% CI: 2.28-15.77; p = 0.00028).

A history of dilatation and curettage was observed more frequently among the cases, as compared to the controls. Nineteen women (63.3%) in the case group had undergone at least one D&C procedure, whereas this history was present in 15 women (25.0%) in the control group. Statistical analysis showed that women with a history of D&C had more than five times greater odds of developing placenta praevia (OR: 5.20, 95% CI: 1.97-13.70; p = 0.00095).

A history of assisted reproductive techniques (ARTs) was also found to be more common among women with placenta praevia. Eighteen cases (60.0%) reported conception following ARTs, compared with 17 women (28.3%) in the control group. Women who conceived with the help of ARTs were almost four times more likely to develop placenta praevia, compared to those who conceived spontaneously (OR: 3.80, 95% CI: 1.49-9.67; p = 0.0037).

Multiple pregnancies were observed in six cases (20.0%) and seven controls (11.7%). Although the ORs suggested a slightly higher risk of placenta praevia in women with multiple pregnancies (OR: 1.90), this association did not reach statistical significance (95% CI: 0.59-6.09; p = 0.29).

A previous history of placenta previa was strongly associated with recurrence in the current pregnancy. Fifteen cases (50.0%) reported a prior placenta previa, compared with only five women (8.3%) in the control group. Women with a history of placenta previa had 11 times higher odds of developing the condition again (OR: 11.0, 95% CI: 3.23-37.42; p = 0.0001), highlighting it as one of the most significant risk factors in our study (Table 1).

**Table 1:** Association between different maternal risk factors and placenta previa Advanced maternal age, previous cesarean section, history of dilatation and curettage, assisted reproductive techniques (ARTs), and a prior history of placenta previa were all found to be significantly associated with the condition. Multiple pregnancies, however, did not show a statistically significant relationship.

Risk factors	Cases (total 30)	Control (total 60)	Odds ratios	p-value
Age > 30 years	17 (56.7%)	15 (25.0%)	3.923	0.0064
Previous C-section	18 (60%)	12 (20%)	6.0	0.00037
History of dilatation and curettage	19 (63.3%)	15 (25.0%)	5.2	0.00095
History of ART	18 (60.0%)	17 (28.3%)	3.8	0.0037
Multiple pregnancies	6 (2%)	7 (11.7%)	1.9	0.29
History of placenta previa in previous pregnancy	15 (50.0%)	5 (8.3%)	11.0	0.0001

## Discussion

In this study, several maternal and obstetric factors were found to be significantly associated with placenta previa. Our findings are consistent with those reported by Latif et al. in a matched case-control study, which demonstrated a significant association between placenta previa and advanced maternal age, previous cesarean section, and prior uterine instrumentation, such as dilatation and curettage. The similarity in results across different populations reinforces the role of uterine scarring and altered endometrial receptivity in the pathogenesis of placenta previa [20], highlighting the multifactorial nature of this condition. Among these, a history of previous cesarean section emerged as one of the most important risk factors. This finding supports the hypothesis that uterine scarring resulting from cesarean delivery may disrupt the normal endometrial-myometrial interface, predisposing to abnormal placental implantation in subsequent pregnancies. Similar observations have been reported previously, including an increased risk of placenta praevia when conception occurs within one year of a cesarean section (risk ratio (RR): 1.7; 95% CI: 0.9-3.1) [21]. Additionally, an elevated risk has been demonstrated in women with a history of prelabour cesarean section in subsequent deliveries (OR: 2.62; 95% CI: 1.24-5.56) [22]. The consistency of our findings with these studies underscores the importance of prior uterine surgery as a key determinant in the development of placenta previa and emphasizes the need for vigilant antenatal surveillance in such women.

ARTs were also significantly associated with placenta previa in our cohort. This association may be explained by underlying uterine or endometrial abnormalities commonly present in women requiring ARTs, as well as altered implantation dynamics related to embryo transfer procedures. Our findings are in agreement with a 2016 meta-analysis of singleton pregnancies that reported a substantially increased risk of placenta praevia (RR: 3.71; 95% CI: 2.67-5.16). This association was further supported by a subsequent 2017 meta-analysis, which demonstrated a similarly elevated odds of placenta praevia (OR: 2.67; 95% CI: 2.01-3.34) [23]. These comparisons reinforce the relevance of ARTs as an emerging and clinically significant risk factor that should be considered during antenatal risk stratification.

In contrast, no significant association between placenta previa and multiple pregnancies was observed in our study. This finding may be attributed to the relatively small sample size and the low number of multiple gestations included in the analysis. However, existing literature suggests a different trend. A large retrospective cohort study involving 67,895 singleton and twin pregnancies demonstrated a higher risk of placenta praevia in twin gestations compared with singleton pregnancies, with an increased risk observed in both dichorionic (OR: 1.54) and monochorionic twins (RR: 3.29) [24]. The discrepancy between our findings and those of larger studies highlights the limitations of smaller single-center studies and suggests that further large-scale research is required to clarify the role of multiple pregnancies in the development of placenta previa.

Advanced maternal age (>30 years) was another factor significantly associated with placenta previa in our study. This finding is consistent with previously published evidence indicating that older maternal age is associated with an increased risk of abnormal placental implantation. Proposed mechanisms include age-related endometrial changes, cumulative exposure to obstetric or gynecological interventions, and vascular alterations, such as atherosclerotic or infarctive changes within the uterine lining [25]. The identification of advanced maternal age as a significant risk factor in our population highlights the need for early diagnosis and careful antenatal monitoring in older pregnant women to minimize maternal and fetal complications.

Despite providing valuable insights, this study has certain limitations. The relatively small sample size and single-center design may limit the generalizability of the findings. Additionally, some potential risk factors, such as smoking and detailed socioeconomic variables, could not be adequately evaluated due to their low prevalence or incomplete documentation. Recall bias and reliance on medical records may also have influenced the accuracy of some variables. Future multicenter studies with larger sample sizes and more comprehensive data collection are recommended to further validate these findings and enhance understanding of the risk factors associated with placenta previa.

## Conclusions

This study identifies several important risk factors associated with placenta previa, including advanced maternal age, previous cesarean delivery, prior dilatation and curettage, ARTs, and a history of placenta previa. Recognizing these factors enables healthcare providers to identify women at higher risk and ensure close monitoring during the antenatal period to minimize complications. Although our findings are consistent with previous research, they add valuable insight by examining these associations in our local population through a focused case-control design.

## Appendices

Appendix 1

Proforma:

Associated Risk Factors of Placenta Previa: A matched case-control study

Medical Record Number:

I) Age Groups:

1.       <20 years of age

2.       20-35 years of age

3.       30-35 years of age

4.       >35 years of age

5.       >40 years of age

II) Parity of patient:

1.       Primigravida

2.       Low parity

3.       Multiparity

4.       Grandmultiparity

III) Previous cesserian section:

1.       One cesarean section

2.      Two Cesarean sections

3.       Three cesarean sections

4.       Four cesarean sections

5.       Five or more cesarean sections

IV) History of placenta previa:

1.       Present

2.       Absent

V) History of multiple pregnancy:

1.       Present

2.       Absent

VI) History of dilation and curettage:

1.       Present

2.       Absent

VII) History of artificial reproductive techniques:

1.       Present

2.       Absent
